# Bionic sensing system and characterization of exhaled nitric oxide detection based on canine olfaction

**DOI:** 10.1371/journal.pone.0279003

**Published:** 2022-12-19

**Authors:** Pengjiao Sun, Yunbo Shi, Yeping Shi

**Affiliations:** 1 The Higher Educational Key Laboratory for Measuring & Control Technology and Instrumentation of Heilongjiang Province, Harbin University of Science and Technology, Harbin, China; 2 Electronics and Communication Engineering School, Jilin Technology College of Electronic Information, Jilin, China; 3 Heilongjiang Province Key Laboratory of Laser Spectroscopy Technology and Application, Harbin University of Science and Technology, Harbin, China; 4 National Experimental Teaching Demonstration Center for Measurement and Control Technology and Instrumentation, Harbin University of Science and Technology, Harbin, China; Tongji University, CHINA

## Abstract

A quantitative monitoring system for fractional exhaled nitric oxide (FENO) in homes is very important for the control of respiratory diseases such as asthma. To this end, this paper proposes a small bionic sensing system for NO detection in an electronic nose based on analysis of the structure of the canine olfactory system and the airflow pattern in the nasal cavity. The proposed system detected NO at different FENO concentration levels with different bionic sensing systems in the electronic nose, and analyzed the data comparatively. Combined with a backpropagation neural network algorithm, the bionic canine sensing system improved the recognition rate for FENO detection by up to 98.1%. Moreover, electronic noses with a canine bionic sensing system can improve the performance of trace gas detection.

## 1. Introduction

Asthma is the most prevalent chronic respiratory disease and affects approximately 235 million people globally [1, 2]. Although incurable, it can be effectively controlled through prevention and treatment [3, 4]. Therefore, the early diagnosis of asthma is crucial for treating it. Human fractional exhaled nitric oxide (FENO) is a biomarker of airway inflammation [5]. According to the *Guidelines for the Clinical Use of eNO*, developed jointly by the ATS/ERS of Europe and USA [6], the amount of FENO in normal adults is <25 ppb (<20 ppb for pediatrics), whereas that in asthmatic patients is >50 ppb (>35 ppb for pediatrics). Prompt detection of FENO can enable the non-invasive diagnosis of asthma.

At present, quantitative testing for asthma markers is only available in hospitals under the supervision of professional medical staff [7]. However, some asthma patients are not fully aware of their own symptoms and their worsening condition [8, 9]. Therefore, there is a need to develop a home-friendly quantitative monitoring system that allows patients to compare symptoms and monitor results daily, so as to control exacerbation. Conventional exhaled gas detection technologies primarily include gas chromatography, mass spectrometry, gas chromatograph-mass spectrometer, electronic nose detection, optics-based breath analyzer, and exhaled gas condensate detection [10–14].

Electronic nose detection ensures simple operation, fast response, short sensor recovery time, low cost, and easy miniaturization. However, it has problems such as low accuracy and poor stability in trace gas detection [15–18]. Therefore, this study was conducted to design a NO detection system to improve the accuracy of trace gas detection in electronic noses.

Electronic noses are designed based on the biotic olfactory sensing system [19]. The olfactory functions of organisms mainly rely on the structure of the nasal cavity, number of olfactory cells, and pattern of nasal airflow. Accordingly, the optimization of the detection performance of the electronic nose mainly focuses on the improvement of the sensor array [20, 21], the pattern recognition algorithm [22, 23], and the structure of the electronic nose [24]. Among them, the electronic nose structure has a direct impact on the diffusion pattern of gas molecules, and thereby is more important for ppb-level gas detection.

Much research has been conducted on the bionic simulation of nasal flow channels, including studies on the electronic nasal cavity with the structure of the dual-channel odor separation column [25] and the fluid dynamics of the detection chamber [26]. Canines exhibit high olfactory performance and their highly developed olfactory recesses render unique nasal airflow patterns [27], which are a key factor in the diffusion and retention of gas molecules in the olfactory region [28]. Therefore, by imitating the structures of olfactory recess, nasal septum, and ethmoturbinate in the canine nasal cavity, the airflow pattern in the bionic nasal cavity of electronic noses can be altered, hence providing a research basis for improving the accuracy of FENO detection in electronic nose systems.

In this study, three biomimetic gas collection cavity structures were developed based on the analysis of the structure of the canine olfactory system and the airflow pattern in the nasal cavity. In addition, the data of NO detected by an electronic nose under three FENO concentration ranges were compared and analyzed. The results verified that electronic noses with canine bionic sensing structures can enhance the detection performance for trace gases, which may facilitate the economical and household application of healthcare equipment such as FENO detectors.

## 2. Design of canine olfactory systems

### 2.1 Structure of electronic nose

**Fig 1:**
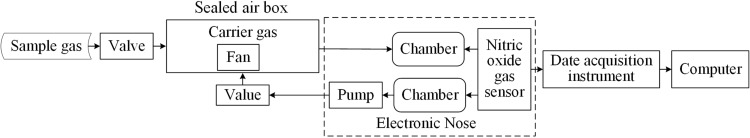
Block diagram of electronic nose detector.

Fig 1 presents a schematic of an electronic nose. It is composed of a NO sensor, electronic nose chambers, an air pump, a fan, a data acquisition card, and a computer. Based on the concentration of exhaled NO gas, the system adopts a sensor produced by NESENSOR (type: 7NE/NO-1), which has a detection resolution and range of 1 ppb and 1–1000 ppb, respectively.

### 2.2 Optimal design of a sensing system mimicking canine in electronic noses

#### 2.2.1 Canine olfactory patterns

The posterior region of the canine nasal cavity is the olfactory recess. According to the trajectory of neutral buoyancy particles in the canine nostril during inhalation [27], the flow paths of breathing air and odorant air in the canine nasal cavity differ. During inhalation, the olfactory airflow passes through the nasal cavity and reaches the olfactory recess, whereas odorant air still fills the olfactory recess during exhalation, increasing the residence time for odor absorption.

#### 2.2.2 The canine-mimicking nasal sensing system

**Fig 2:**
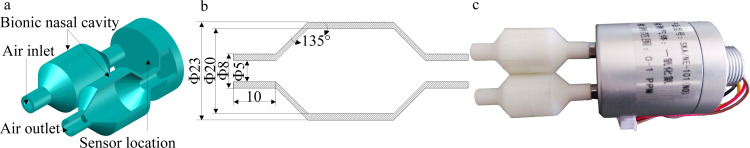
General cavity of the electronic nose system for bionic canine olfaction. (a) Structural diagram of Chamber A. (b) Longitudinal section of Chamber A. (c) Actual picture of Chamber A.

The olfactory functions of canines rely on their unique nasal airflow patterns, which are formed by the olfactory recess [27]. Based on this theory and investigations related to electronic nose cavities [29], a cylindrical dual-chamber structure featuring “a large chamber with a small inlet,” named Chamber A, was adopted in the proposed cavity. The sensor was placed in the position of the “olfactory recess,” forming a dual-chamber sensing system mimicking the canine nasal airway. The diameter of the gas inlet and outlet was 5 mm. Its basic structure is shown in Fig 2, which mainly comprises an air inlet, air outlet, bionic nasal cavity, and airflow channel. Fig 2A is the structural diagram of chamber A; Fig 2B is its longitudinal sectional view, and Fig 2C is its actual picture.

#### 2.2.3 The canine-mimicking nasal septum sensing system

**Fig 3:**
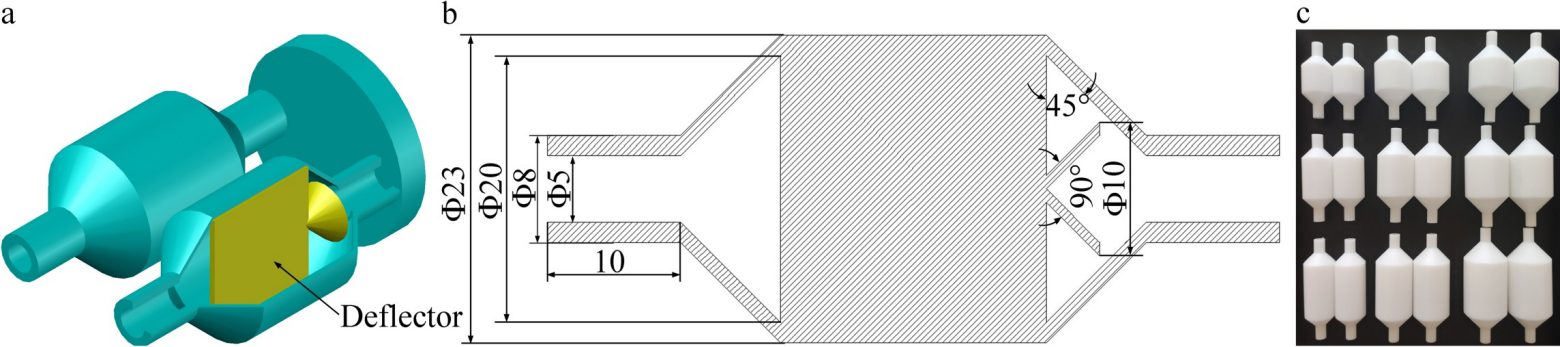
Structure of sensing system mimicking canine nasal septum. (a) Structural diagram of Chamber B. (b) Longitudinal section of Chamber B. (c) Actual picture of Chamber B.

According to the structural characteristics of the vertical plate in the canine nasal septum and ethmoid bone [30–32], a deflector with a thickness of 1 mm was added based on Chamber A in the electronic nose. A hollow cone was placed at the center of the deflector end (Fig 3). This chamber structure is named Chamber B.

#### 2.2.4 The canine-mimicking ethmoid turbinate sensing system

The olfactory recess in the canine nasal cavity is located at the rear of the nasal cavity, which contains a scroll-shaped ethmoturbinate [32, 33]. The surface of ethmoturbinate is covered with olfactory epithelium, where the olfactory nerves and olfactory sensory cells are distributed [34]. In the ethmoturbinate, the olfactory airflow leaves the nasal cavity or flows into the ethmoturbinate extension on the most dorsal side of the olfactory recess after being filtered by the airway labyrinth [27].

**Fig 4:**
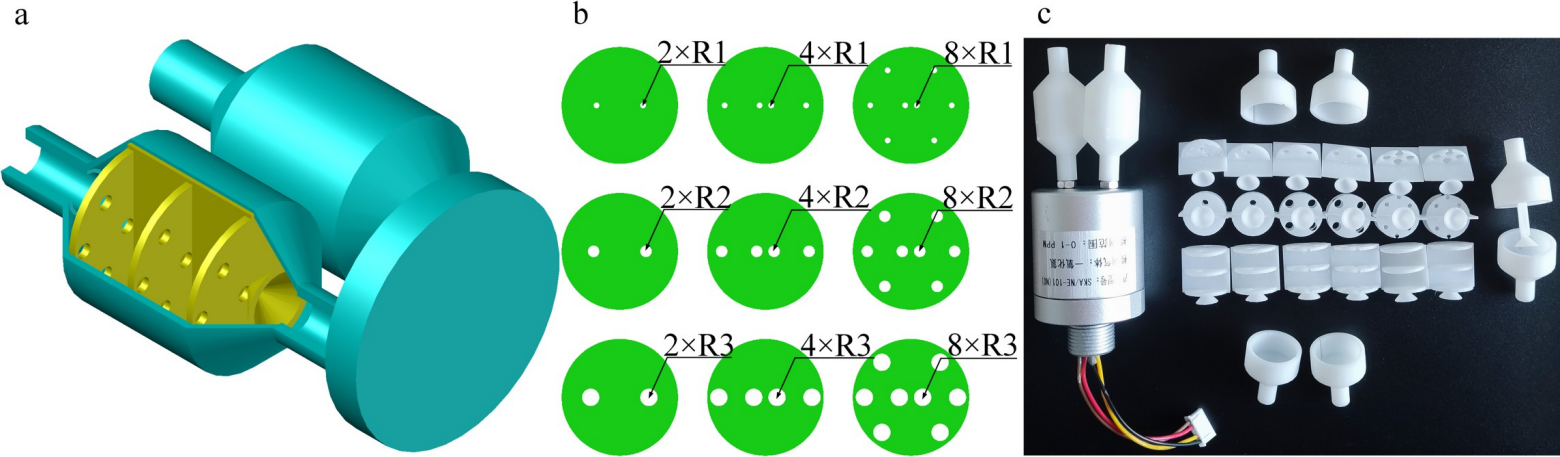
Sensing system mimicking canine ethmoturbinate. (a) Structural diagram of Chamber C. (b) Sieve plates of mimicking ethmoturbinate. (c) Actual picture of Chamber C.

Based on the characteristics of this airway labyrinth in ethmoturbinate, 1–3 sets of sieve plates were introduced to Chamber B of the electronic nose. The sieve plates were placed at the front, middle, and rear of the chamber (Fig 4); they were 1 mm thick with a center spacing of 10 mm between sieve plates. This bionic electronic nose chamber designed by mimicking canine ethmoturbinate is named Chamber C.

## 3. Experiment and simulation

### 3.1 Experimental equipment

**Fig 5:**
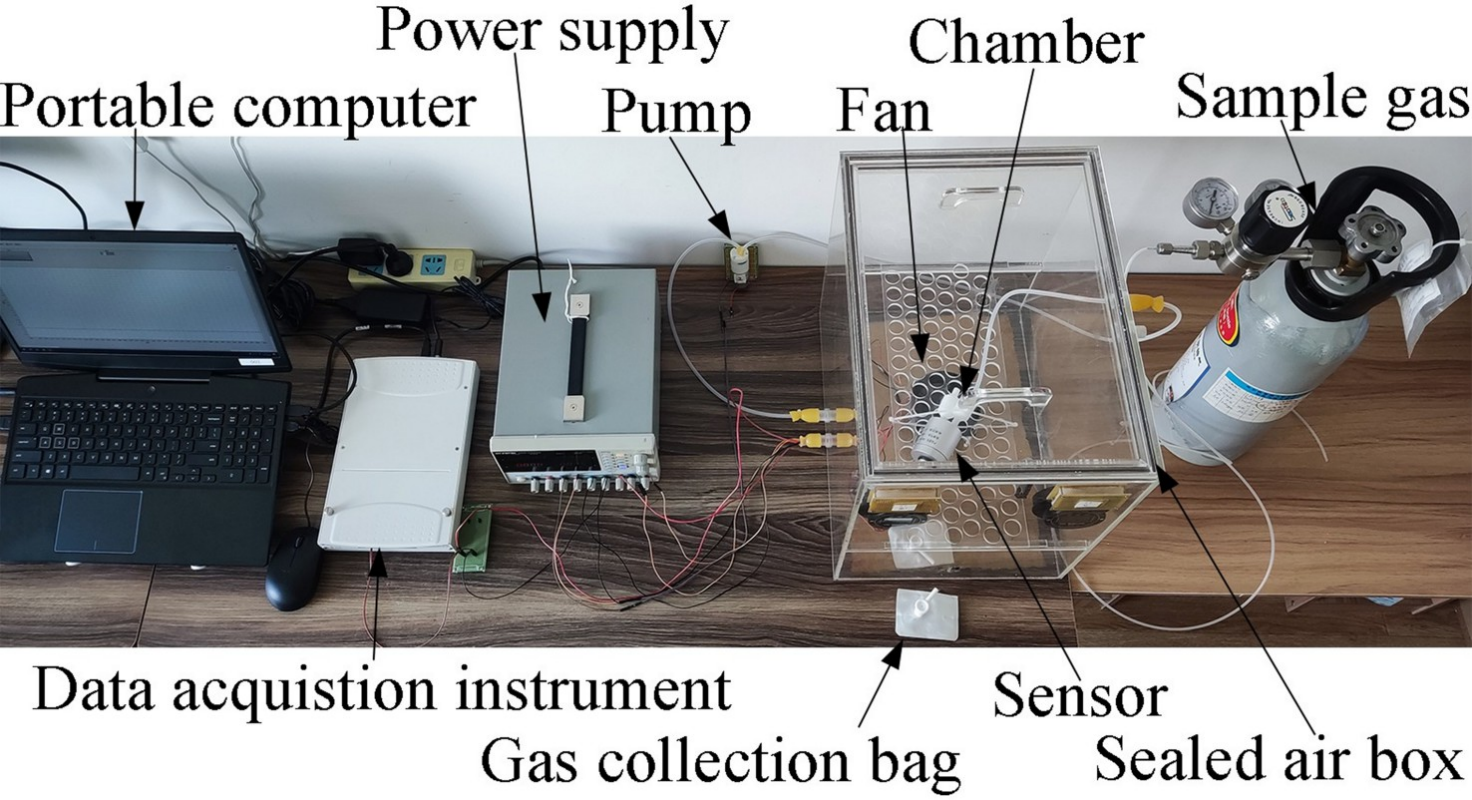
Experimental electronic nose detection system.

**Fig 6:**
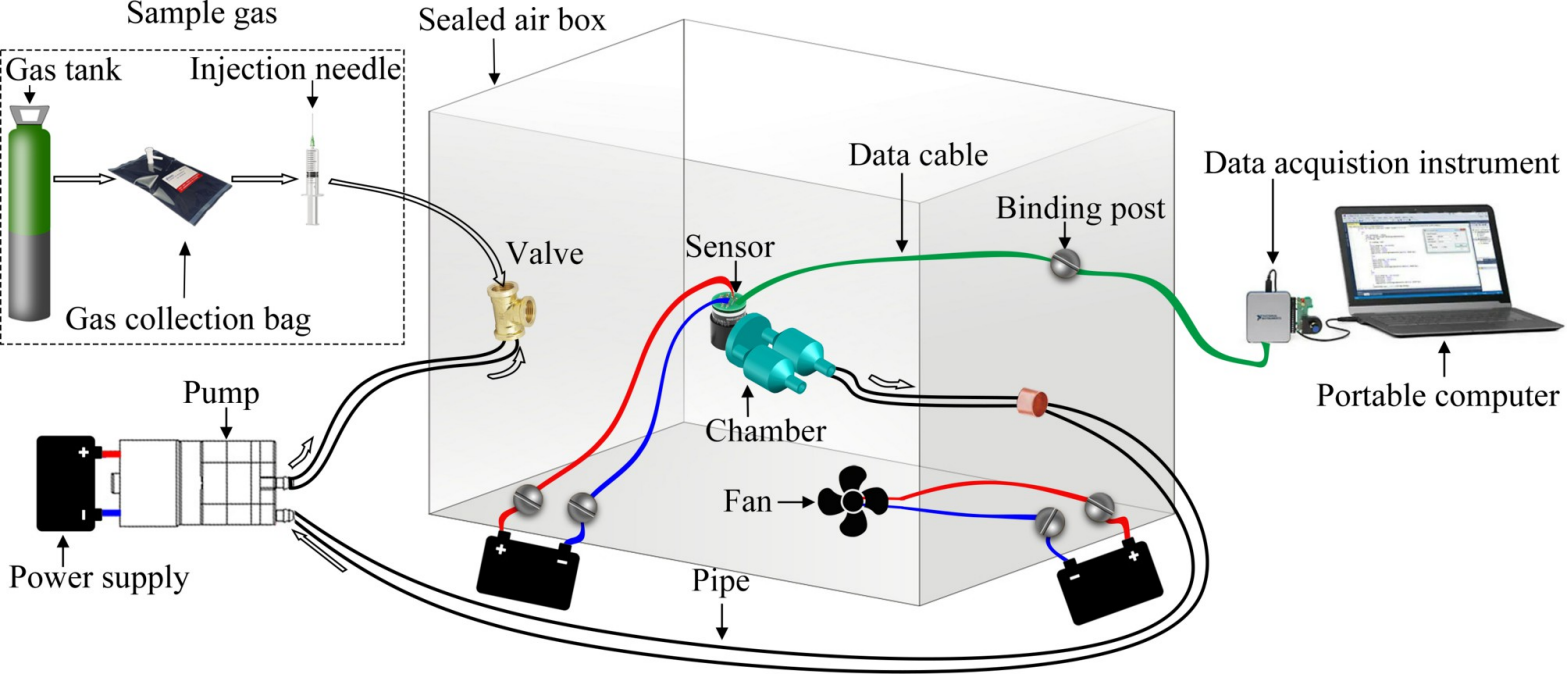
Schematic diagram of experimental electronic nose detection system.

The electronic nose system adopted in the experiment is illustrated in Figs 5 and 6; in this system, the National Instruments USB6289 data acquisition card was used. Moreover, a micro diaphragm air pump (EDLP600-D12B) produced by KAMOER was employed, and its suction flow was adjusted by the voltage input in this experiment. The three bionic cavities used in the experiment were fabricated with Zrapid SLA880, industrial-grade light-curing 3D printing equipment. The material used was white photosensitive resin with a tolerance of 0.1 mm and arc smoothness of 1000. Sampling bags made of Teflon FEP fluorine membrane, manufactured by Ningbo Hongpu Experimental Technology, were adopted as gas collection bags. The volume of the sealed air box was 48 L (0.3 × 0.4 × 0.4 m). The standard gas sample contained a mixture of nitrogen and nitric oxide, obtained from Harbin Liming Gas, with a NO concentration of 2050 ppm.

### 3.2 Experimental procedure

#### 3.2.1 Optimal parameter selection for the canine-mimicking nasal cavity

The parameters of the canine-mimicking nasal cavity of the electronic nose were optimized through orthogonal experiments. An L9 (34) factor-level table of four factors and three levels for orthogonal experiments was developed (Table 1) to investigate the length (A), inner diameter (B), pump suction flow (C), and error grade (D) of bionic Chamber.

**Table 1:** Factors and levels for the canine-mimicking nasal cavity.

Level	Factor			
	A (mm)	B (mm)	C (L/min)	D
**1**	20	20	0.5	1
**2**	30	25	1	2
**3**	40	30	0.5	3

#### 3.2.2 Selection of optimal parameters for sieve plate structure and size

The parameters of the sieve plate structure and size for the electronic nose were optimized via orthogonal experiments. An L9 (34) factor-level table of four factors and three levels was developed for the orthogonal experiments (Table 2) to investigate the number of sieve plates (E), number of pores on sieve plates (F), pore diameter (G), and error grade (H) in Chamber C.

**Table 2:** Factors and levels for the sieve plate structure and size.

Level	Factor			
	E	F	G (mm)	H
**1**	1	2	1	1
**2**	2	4	2	2
**3**	3	8	3	3

#### 3.2.3 Detection experiments of three bionic sensing systems

To verify the detection performance of the electronic nose for the proposed bionic chambers, different concentrations of NO gas were detected, and the response voltage signals were recorded. Gas from the standard sample was collected using gas collection bags; subsequently, NO gas (with a volume of 0.35, 0.7, and 1.05 ml) was injected into the sealed gas chamber; after diffusion, the NO concentration was found to be 15, 30, and 45 ppb, respectively. The response voltage of the sensor was recorded for 60 s in different bionic chambers and the experiment was repeated 30 times.

### 3.3 Computational fluid dynamics (CFD) simulations

The bionic chamber model was imported to the ANSYS software; the internal structure of the bionic chamber was captured by SpaceClaim. The bionic chamber’s inlet pipe has an inner diameter of 5 mm and a length of 10 mm. The pipe in the center of the chamber has an inner diameter of 20 mm and a length of 20 mm. The height of the hollow frustum of a cone between the two pipes is 7.5 mm. The chamber is a symmetrical structure. The center distance between the two chambers is 24 mm. The hollow cylinder at the chamber’s end measures 30 mm in diameter and 3 mm in height. The height of the hollow cone in Chamber B is 4 mm. The center distance between two sieve plates in Chamber C is 10 mm, with one sieve plate in the chamber’s front and the other in the chamber’s center. The model structure was meshed into approximately 600,000 grids. According to the boundary conditions set in the previous experiment, the pump suction flow was 1 L/min with a flow rate of -0.85 m/s at the chamber inlet. The shear-stress transport k-ω model was selected for the calculation. Eventually, ANSYS Fluent was adopted to perform CFD analysis on the internal airflow in the chamber of the electronic nose.

## 4. Results

### 4.1 Experimental results of optimal parameters for the canine-mimicking nasal cavity

When the NO concentration in the sealed box was 200 ppb, the response voltage of the electronic nose was averaged using MATLAB. The mean voltage is shown in S1 Table. In addition, variance analysis and significance testing were performed on these factors using the SPSS software to acquire results after calculation (Table 3). According to these results, the order of influence of factors was determined to be A > C > B, and the optimal combination of detection conditions was A1B1C2.

**Table 3:** Data analysis results for optimal parameters for canine-mimicking nasal cavity.

Source of difference	Range (V)	Mean square	F value
**A**	0.285	0.080	4.249
**B**	0.192	0.029	1.516
**C**	0.211	0.350	1.837

### 4.2 Experimental results for optimal parameters for sieve plate structure and size

When the NO concentration in the sealed box was 200 ppb, the response voltage of the electronic nose was averaged using MATLAB. The mean voltage is shown in S2 Table. In addition, variance analysis and significance testing were performed for the factors using the SPSS software (Table 4). According to the results obtained, the order of influence of factors was found to be G > E > F, and the optimal combination of detection conditions was E2F3G1.

**Table 4:** Data analysis results for optimal parameters for sieve plate structure and size.

Source of difference	Range (V)	Mean square	F value
**E**	0.112	0.010	1.827
**F**	0.102	0.008	1.490
**G**	0.144	0.019	3.363

### 4.3 Response of three bionic sensing systems

In the experiment, parameters such as the size of the bionic chambers and the pumping flow rates were obtained from the experimental results in Sections 4.1 and 4.2. The inner diameter of the cavity was 20 mm, and its length was 20 mm. There were two sieve plates in Chamber C, with eight holes of 1 mm in diameter in each plate, and the pump flow rate of the electronic nose sensing system was 1 L/min.

**Fig 7:**
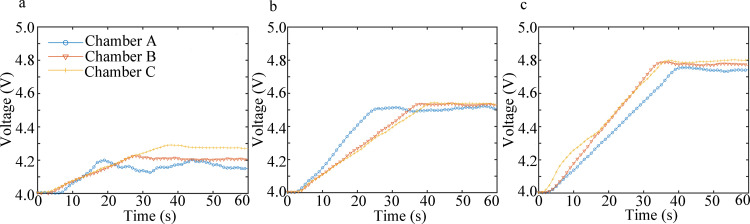
Voltage response mean plots of the three bionic sensing systems under three NO gas concentrations: (a) 15 ppb, (b) 30 ppb, and (c) 45 ppb.

**Fig 8:**
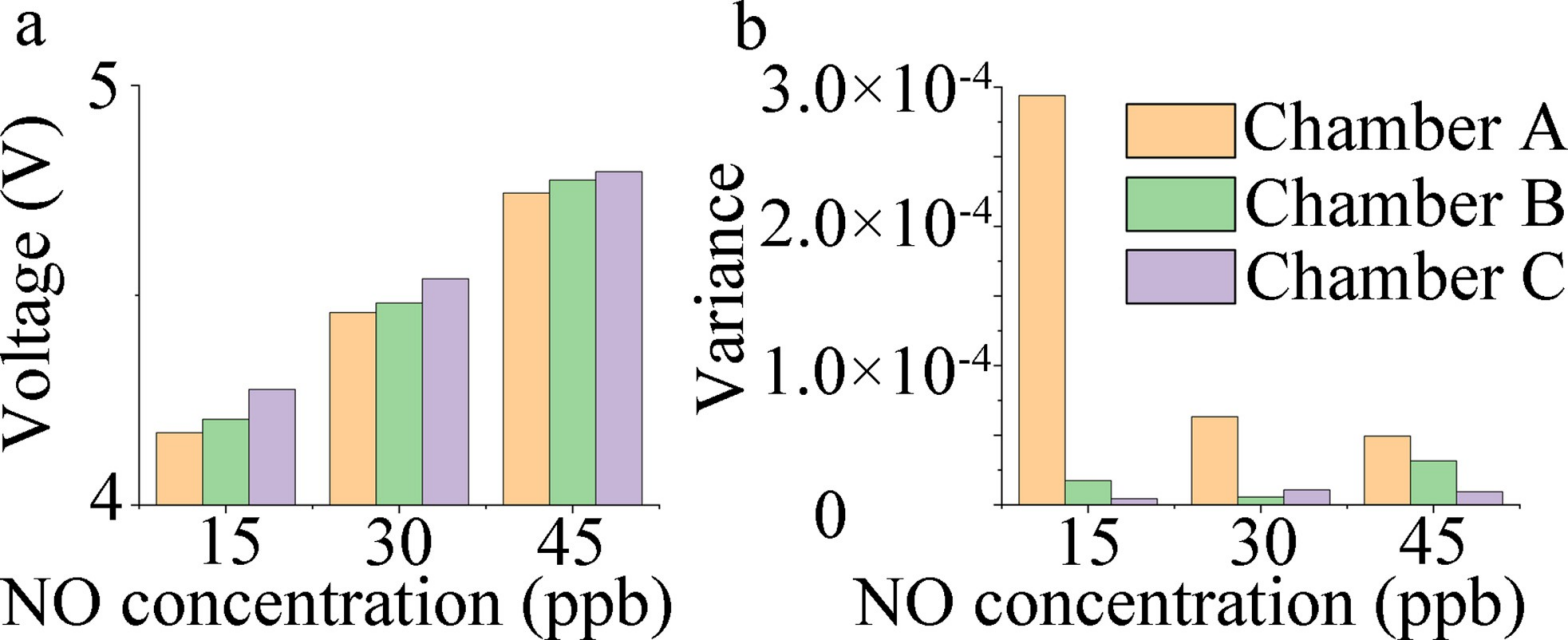
Histograms of mean and variance of sensor response voltage. (a) Voltage response mean of the sensor under the three NO gas concentrations. (b) Variance of sensor response voltage at the three NO gas concentrations.

The response voltage data of the electronic nose were smoothed using MATLAB. Moving average was adopted as the smoothing method, with a smoothing factor of 0.5. The acquired results are illustrated in Fig 7. The mean and variance of the voltage signal for 40–60 s for each group are illustrated in Fig 8 (S3 and S4 Tables). At a constant NO concentration, the electronic nose with the Chamber C sensing system mimicking canine ethmoturbinate exhibited the smallest detection error, as demonstrated in Table 5.

**Table 5:** Errors of the three bionic sensing systems.

NO concentration	Chamber A	Chamber B	Chamber C
**15 ppb**	1.05 ppb	0.93 ppb	0.69 ppb
**30 ppb**	1.35 ppb	1.24 ppb	1.14 ppb
**45 ppb**	1.94 ppb	1.67 ppb	1.49 ppb

### 4.4 Response recognition of electronic nose based on backpropagation (BP) algorithm

**Fig 9:**
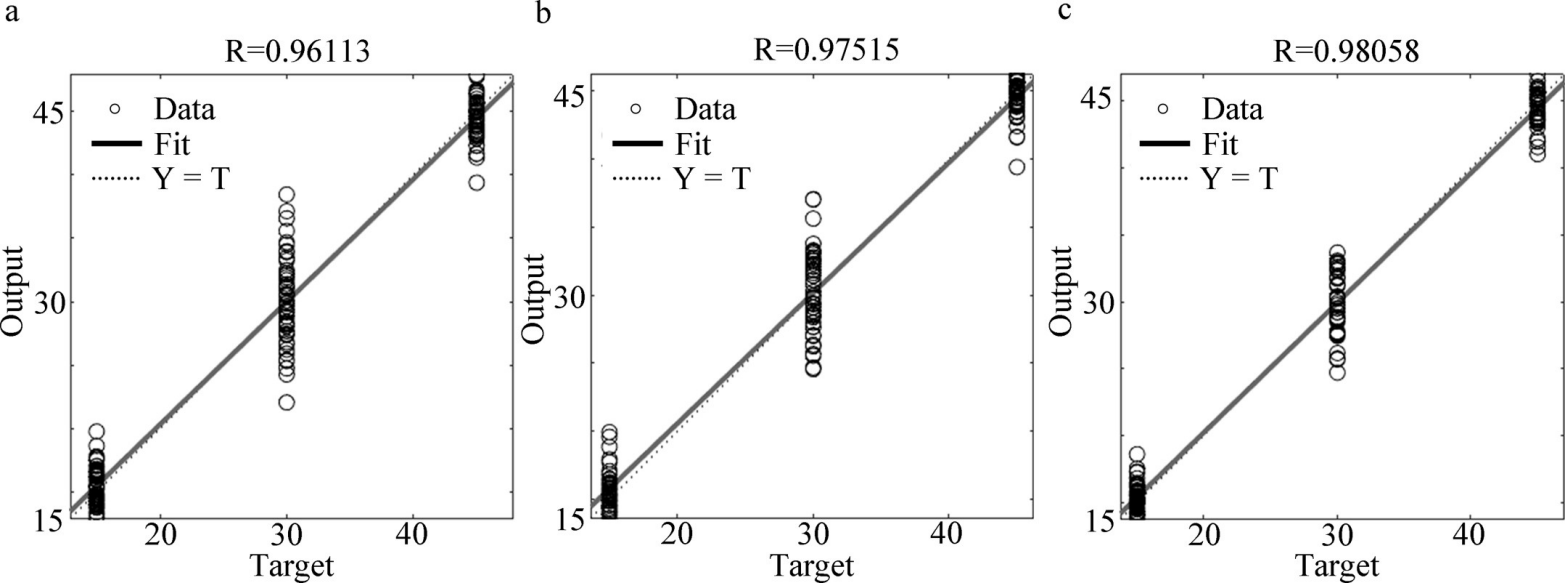
Comprehensive results of training electronic nose response by the backpropagation (BP) neural network. (a) Sensing system of Chamber A. (b) Sensing system of Chamber B. (c) Sensing system of Chamber C.

A BP neural network was trained with the Neural Network Fitting app in MATLAB to establish a recognition model for the electronic nose system. The voltage signal for each group was classified from 40–60 s. A total 360 valid datasets were divided into 252 training, 54 verification, and 54 test datasets while imposing one hidden layer. The comprehensive results of these datasets are summarized in Fig 9. The errors in the training and verification datasets were found to be less than 4%. As demonstrated in Fig 9, Chamber C exhibited the optimal training effect (up to 98.1%).

### 4.5 CFD simulation results

**Fig 10:**
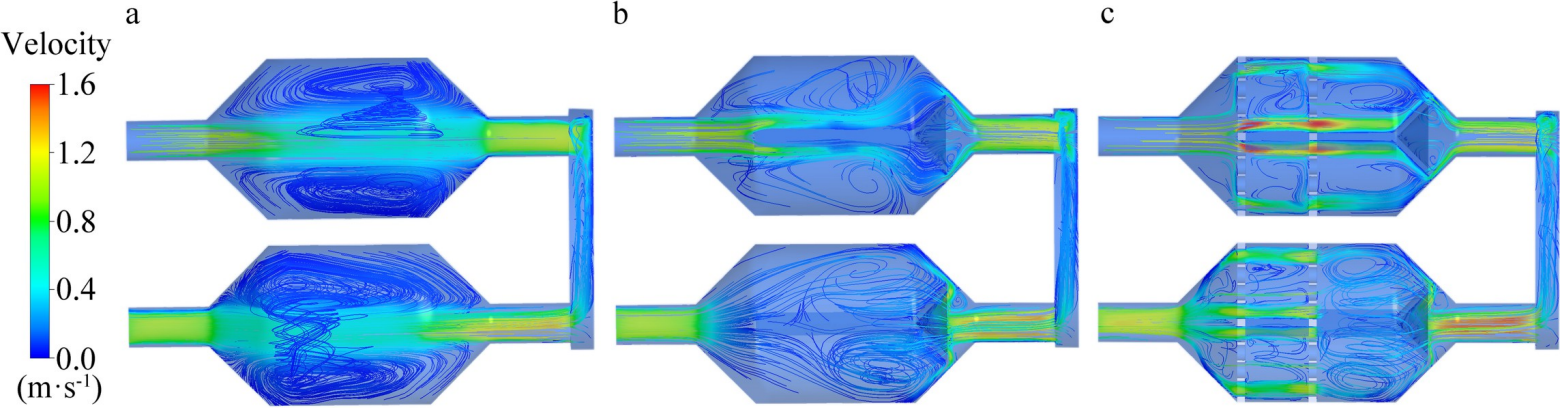
Airflow velocity profile of the three sensing systems. (a) Maximum flow rate in Chamber A was 1.38 m/s. (b) Maximum flow rate in Chamber B was 1.42 m/s. (c) Maximum flow rate in Chamber C was 1.58 m/s.

As illustrated in Fig 10, the simulation results explain the velocity profile in the chamber of the sensing systems. It can be seen that the three chamber structures produced different airflow patterns under the same boundary conditions. The turbulent flow in Chamber A was a large-scale vortex. The septum in Chamber B evenly divided the air passage into two parts, where the inhaled gas was parted and fully mixed, generating a small-scale vortex, and the airflow passed steadily through the area under sensing. In Chamber C, vortices of sizes smaller than those in chamber B were generated as it went through the sieve plate.

The highest flow rate in each chamber was vA = 1.38 m/s, vB = 1.42 m/s, vC = 1.58 m/s, respectively. The gas flow velocity in Chamber C was the highest, located at the air hole in the sieve plate and rear of the bionic chamber. The posterior region was the sensor perception area, mimicking the olfactory recess of the canine nasal cavity, where the airflow rate was relatively fast, and the sensor could fully contact the multiple currents generated, playing an important role in improving the detection accuracy and sensitivity of the sensor.

## 5. Discussion

Sensing systems with a dual-chamber design mimicking the nasal cavity of canines can enhance the detection performance of electronic noses at low FENO concentrations. In this work, an independent dual-chamber structure was designed to simulate the biotic exhalation and inhalation processes. According to the characteristics of the canine nasal structure and nasal airflow, the sensing system designed by mimicking the canine ethmoturbinate demonstrated optimal detection performance.

As demonstrated in Fig 8, during the detection of trace FENO with three different gas concentrations (15, 30, and 45 ppb), the sensing system of Chamber C yielded the largest average response voltage, lowest variance, and best detection stability, while that of Chamber A yielded the smallest average response voltage, highest variance, and poorest detection stability. As shown in Table 5, the detection error of the Chamber C sensing system was lower than that of Chambers B and A. For the three gas concentrations, the minimum error was 4.6%, 3.8%, and 3.3%, respectively, and the Chamber C sensing system exhibited the highest detection accuracy.

As illustrated in Fig 9, all errors for electronic nose detection in the bionic sensing systems were less than 4% after the response data were processed using the BP neural network. Moreover, in comparison to response data without data processing, this procedure reduced the detection error of and improved the detection accuracy of the electronic nose detection system. Chamber C demonstrated the optimal training effect, where a recognition rate of 98.1% could be achieved.

As shown in Fig 10, Chamber C with added sieve plates exhibited the highest gas flow velocity; the region with the maximum flow velocity was located at the pores of the sieve plates and rear of the chamber. Distinct small-scale vortices were generated in the gas flow, which increased the time of gas diffusion and retention in the sensing area. Craven et al. [27] reported that small vortices may be generated in the airflow at the olfactory area of mammalian nasal cavities, thus rendering more sufficient contact between gas molecules and olfactory cells. Hence, gas molecules can thoroughly contact the sensor surface by producing air vortices at the surface, thereby enhancing the detection performance of the electronic nose system. This conclusion was further confirmed by the CFD calculation results.

Our discoveries are consistent with previous investigations on the bionic chamber of electronic noses [29, 35]. Based on the bionic theory, the performance of electronic nose detection systems can be enhanced by altering the chamber structure of the electronic nose.

In this work, an innovative sensing system with a dual-chamber electronic nose based on the structure of canine ethmoturbinate was designed. To enhance the detection performance, canine nasal structures were analyzed and the sensor was placed at the “olfactory recess” with high vortex intensity, i.e., the rear of the chamber. According to the characteristics of nasal airflow patterns and olfactory airway labyrinth in canine nasal cavities, airflow sieve plates were designed based on the bionic theory. Under the effects of airflow sieve plates, the vortices generated in the airway can extend the diffusion and retention time of target gas molecules at the sensing area, thereby enhancing the accuracy and stability of the sensor for trace gas detection.

However, this study only verified and analyzed the data at three concentration levels as detected by a single sensor, without considering the influence of different temperatures and humidities on the detection results. In subsequent research, human FENO detection experiments will be carried out, and a temperature and humidity compensation model will be established to compare the performance of the analysis and detection systems of the medical equipment. This study provides a basis for the design of FENO quantitative monitoring equipment for household use. Moreover, it provides an experimental basis for enhancing the performance of electronic nose systems for trace gas detection.

## 6. Conclusions

Based on the features of FENO detection, structure of canine olfactory systems, and airflow patterns in olfactory airways, a bionic chamber structure for gas collection that mimics the canine nasal structure was proposed in this study. The bionic sensing systems with electronic noses were fabricated via 3D printing. Moreover, the NO data detected under three FENO concentration ranges were compared and analyzed. The results showed that the sensing system with an independent dual-chamber structure mimicking the canine ethmoturbinate achieved the highest detection stability and smallest detection error. The combination of bionic sensing system and BP neural network further mitigated the detection error and increased the recognition rate of the electronic nose detection system up to 98.1%. This indicates that electronic noses with canine bionic sensing systems can enhance trace gas detection performance, thus satisfying the functional and technical requirements of economical and miniaturized FENO detectors for household users with asthma. Furthermore, it provides a research basis for the development of household FENO quantitative monitoring equipment.

## Supporting information

S1 Table Test results of sensing system mimicking canine nasal. (XLSX)

S2 Table Test results of Sensing system mimicking canine ethmoturbinate. (XLSX)

S3 Table Mean value. (XLSX)

S4 Table Variance value. (XLSX)

S5 Table Sensor response voltage of experiment 1. Experiment of optimal parameter selection for the canine-mimicking nasal cavity.
(XLSX)

S6 Table Sensor response voltage of experiment 2. Experiment of selection of optimal parameters for sieve plate structure and size.
(XLSX)

S7 Table Sensor response voltage of experiment 3. Detection experiments of three bionic sensing systems.
(XLSX)
